# Polymorphisms in Genes of Tricarboxylic Acid Cycle Key Enzymes Are Associated with Early Recurrence of Hepatocellular Carcinoma

**DOI:** 10.1371/journal.pone.0124471

**Published:** 2015-04-20

**Authors:** Shaogui Wan, Yousheng Wu, Xingchun Zhou, Yibing Chen, Jiaze An, Xiaohe Yu, Huiqing Zhang, Hushan Yang, Jinliang Xing

**Affiliations:** 1 Institute of Pharmacy, Pharmaceutical College of Henan University, Kaifeng, Henan, China; 2 State Key Laboratory of Cancer Biology, Experimental Teaching Center of Basic Medicine, Fourth Military Medical University, Xi'an, Shaanxi, China; 3 Department of Hepatobiliary Surgery, Xijing Hospital, Fourth Military Medical University, Xi'an, Shaanxi, China; 4 Department of Interventional Radiology, Eastern Hepatobiliary Surgery Hospital, Second Military Medical University, Shanghai, China; 5 Department of Pain treatment, Tangdu Hospital, The Fourth Military Medical University, Xi'an, China; 6 Division of Population Science, Department of Medical Oncology, Kimmel Cancer Center, Thomas Jefferson University, Philadelphia, Pennsylvania, United States of America; University of Modena & Reggio Emilia, ITALY

## Abstract

Alterations of activity and expression in tricarboxylic acid (TCA) cycle key enzymes have been indicated in several malignancies, including hepatocellular carcinoma (HCC). They play an important role in the progression of cancer. However, the impact of single nucleotide polymorphisms (SNPs) in genes encoding these key enzymes on the recurrence of HCC has not been investigated. In this study, we genotyped 17 SNPs in genes encoding TCA cycle key enzymes and analyzed their association with recurrence-free survival (RFS) in a cohort of 492 Chinese HCC patients by Cox proportional hazard model and survival tree analysis. We identified 7 SNPs in *SDHC*, *SDHD*, *FH*, and *IDH2* genes to be significantly associated with the RFS of HCC patients. Moreover, all these SNPs were associated with the early recurrence (within 2 years after surgery) risk of diseases. Cumulative effect analysis showed that these SNPs exhibited a dose-dependent effect on the overall and early recurrence. Further stratified analysis suggested that number of risk genotypes modified the protective effect on HCC recurrence conferred by transcatheter arterial chemoembolization treatment. Finally, the survival tree analysis revealed that SNP rs10789859 in *SDHD* gene was the primary factor contributing to HCC recurrence in our population. To the best of our knowledge, we for the first time observed the association between SNPs in genes encoding TCA cycle key enzymes and HCC recurrence risk. Further observational and functional studies are needed to validate our findings and generalize its clinical usage.

## Introduction

Hepatocellular carcinoma (HCC) is one leading cause of cancer death worldwide[1]. The current curative therapies for HCC are limited to early stage diseases with first-line treatments of surgical resection and liver transplantation. However, the recurrence and metastasis occur frequently after curative surgical treatment [2]. It has been estimated that the 5-year recurrence rate is frequently higher than 70% in HCC patients with early stage disease after curable treatments such as surgical resection and ablation, although significant low recurrence rate (<15%) can be obtained by treatment with liver transplantation [3]. In current clinical settings, prognosis assessment and recurrence prediction after surgical treatment in HCC patients are mainly based on clinicopathological characteristics, such as stages according to the Barcelona Clinic Liver Cancer (BCLC) or TNM staging system [3,4]. However, the lack of sensitivity for predicting individual patient’s prognosis led to the necessity for an improved prediction system by introducing tumor biomarkers [5]. Unfortunately, specific biomarkers that can be validated and expended to clinical settings are still lacking and urgently needed.

The tricarboxylic acid (TCA) cycle is a core pathway for the metabolism of sugars, lipids, and amino acids in the mitochondria [6]. The fundamental role of TCA cycle is to constantly oxidize the acetyl moiety of acetyl-CoA to CO_2_ and to generate NADH and FADH_2_ which feed electrons to the respiratory chain for ATP generation [7]. Three enzymes, succinate dehydrogenase (SDH), fumarate hydratase (FH), and isocitrate dehydrogenase (IDH), are well recognized as key players in maintaining the physiological role of TCA cycle inside the mitochondria. Recent studies have suggested that the malfunctions of TCA cycle, especially the abnormally altered activities of three key enzymes caused by the genetic mutations, are associated with many cancer initiation and progression[8–11]. For example, it has been reported that the succinate-producing activity, which is usually used as an indicator for metabolizing activity of TCA cycle in mitochondria, is significantly higher in the HCC tumor tissues than that in non-tumor tissues [11].

Single nucleotide polymorphism (SNP) is the most common form of genetic variations, and emerging evidences have shown that SNPs may be used as promising surrogate biomarkers of individual genetic background to predict therapeutic responses and prognosis in cancer patients. And the candidate individual SNP assay is easy to perform in most of clinical laboratory by probe-based Real-time quantitative PCR method [12]. However, up to date, there is no study to investigate the effect of SNPs in the genes encoding TCA cycle key enzymes on HCC recurrence. In this study, we sought to evaluate the associations between functional SNPs in genes encoding three TCA cycle key enzymes (SDH, FH, and IDH) and HCC recurrence in a hospital-based Chinese patient cohort.

## Materials and Methods

### Study population

The study subjects were recruited from Eastern Hepatobiliary Surgery Hospital affiliated to The Secondary Military Medical University (SMMU) in Shanghai and Xijing Hospital affiliated to The Fourth Military Medical University (FMMU) in Xi’an, China. Between January 2009 and January 2012, a total of 518 Han Chinese patients diagnosed with primary HCC were included in the initial cohort. HCC was diagnosed based on the National Comprehensive Cancer Network clinical practice guidelines in oncology. All patients had no history of other cancers. All patients received surgery within 2 months after diagnosis and without any anticancer treatment before surgery. In this present study, we finally analyzed 492 patients after excluding 26 patients, which including 3 patients with incomplete clinical data and 23 patients died within 1 month post-surgery. This study was approved by the Ethic Committee of FMMU and SMMU, and a signed informed consent was obtained from each participant.

### Data collection and follow-up

Demographic variables including age, gender and clinical data such as serum HBsAg status, serum AFP level, tumor characteristics, and disease stages were collected through medical chart review and consulting with the treating physicians by trained clinical specialists. Patients were followed up 1 month after surgical resection treatment, and the follow-up information was updated at 6-month intervals through onsite interviews, direct calling, or medical chart review. The last follow-up data in this study was obtained in January 2013.

### Selection and genotyping of SNPs

Blood sample (5mL) was obtained from each participant before any treatment for genomic DNA extraction using E.Z.N.A. Blood Midi Kit (Omega Bio-Tek, Norcorss, GA, USA). The candidate functional SNPs in TCA cycle key enzymes were selected by a set of web-based SNP selection tools (http://snpinfo.niehs.nih.gov/snpinfo/snpfunc.htm). The SNP selection criteria were as following: 1) potential functional SNPs should have minor allele frequency ≥ 5% in Han Chinese population in the HapMap database; 2) SNPs should be located in miRNA binding sites of the 3’ untranslated region (3’UTR), in the transcription factor binding site of 5’ untranslated region (5’UTR), in the mRNA splice site, or in exons; 3) If there were multiple candidate SNPs within the same haploid block and the linkage disequilibrium (LD) coefficient r^2^ > 0.8, only one SNP was included. Finally, a total of 17 potential functional SNPs in *SDH* (including subunits of *SDHA*, *SDHB*, *SDHC*, and *SDHD*), *FH*, and *IDH* (including subunits of *IDH1* and *IDH2*) genes were identified. SNPs were genotyped in blood DNA which can represent those from the liver tissues using the Sequenom iPLEX platform (Sequenom Inc., San Diego, CA, USA) and laboratory persons who conducted the genotyping assays were blinded to patients’ information. Quality controls were implemented in each assay for genotyping and SNP with call rate >98% was included for further analysis. The detailed information of candidate SNPs and genotyping results were listed in S1 Table.

### Statistical analysis

The primary endpoint of this study was recurrence-free survival (RFS). Recurrence was defined as the regrowth of tumor in the original organ or different organ after the surgical treatment, which was diagnosed based on the combined examinations of abdominal imaging findings (MRI and CT) and laboratory examinations. RFS was defined as the time from surgical treatment to local recurrence or distant metastasis. All patients without recurrence or lost during the follow-up were censored for the analysis. The secondary endpoints were early recurrence, which occurred within 2 years after surgical treatment, and late recurrence, which occurred more than 2 years after surgery. The association between single SNP and HCC recurrence risk was estimated by hazard ratios (HRs) generated from Cox proportional hazards regression model using multivariate analysis. To reveal whether the significant results in our analysis were false positive due to multiple SNPs were analyzed, the *q* value that reflects the false discovery rate was used to adjust the significance for individual SNP by *q* value package implemented in the R software. Kaplan-Meier survival curve and log-rank test were used to assess the differences in RFS among different patient groups. Cumulative effect was evaluated by the combination of risk genotypes identified from the main analyses of the individual SNPs, in which the risk genotype was defined as that genotype carried by patient group who had significant worse clinical outcomes in the survival analyses. All above mentioned statistical analyses were performed using the IBM SPSS Statistics 19.0 software (IBM). Survival tree analysis was used to determine the higher-order gene-gene interactions using the STREE program (http://masal.med.yale.edu/stree/) in which recursive-partitioning was used to identify subgroups of individuals at higher risk. All *P* values were 2-sided, and *P* < 0.05 was considered as statistical significance.

## Results

### Characteristics of the study population

As shown in Table 1, 295 patients developed recurrence during the median follow-up time of 22.7 months (range, 1.6–48.3 months). There were 60 patients lost during the follow-up duration and censored for further analysis, and the rate of follow-up lost was 12.2%. When compared with those in reference group, there was a significantly higher risk of recurrence in patients with higher serum AFP (*P* = 1.8 × 10^-4^), larger tumor size (*P* = 3.2 × 10^-4^), multiple tumors (*P* = 5.1 × 10^-5^), poor differentiated tumors (*P* = 3.3 × 10^-6^), advanced TNM stages (*P* = 9.9 × 10^-7^) and BCLC stages (*P* = 6.5 × 10^-8^), and port vein tumor thrombus (*P* = 1.5 × 10^-5^).

**Table 1:** Characteristics distribution of study population by recurrence.

Variables	No recurrence (%), n = 197	Recurrence (%), n = 295	*P* value*
Age, mean (SD)	53.5 (10.7)	51.7 (10.7)	0.064
Gender			
Female	33 (16.8)	34 (11.5)	
Male	164 (83.2)	261 (88.5)	0.098
HBsAg			
Negative	19 (9.6)	27 (9.2)	
Positive	178 (90.4)	268 (90.8)	0.854
Serum AFP (ng/ml)			
< 200	126 (64.0)	138 (46.8)	
≥ 200	71 (36.0)	157 (53.2)	1.8 × 10^-4^
Tumor size (cm)			
< 5	103 (52.3)	106 (35.9)	
≥ 5	94 (47.7)	189 (64.1)	3.2 × 10^-4^
Tumor number			
Single	176 (89.3)	220 (74.6)	
Multiple	21 (10.7)	75 (25.4)	5.1 × 10^-5^
Tumor differentiation			
Well and moderate	82 (41.6)	65 (22.0)	
Poor	115 (48.4)	230 (78.0)	3.3 × 10^-6^
TNM stage			
Stage I + II	179 (90.9)	215 (72.9)	
Stage III + IV	18 (9.1)	80 (27.1)	9.9 × 10^-7^
BCLC stage			
A	172 (87.3)	190 (64.4)	
B	15 (7.6)	47 (15.9)	
C	10 (5.1)	58 (19.7)	6.5 × 10^-8^
PVTT status			
Negative	192 (97.5)	247 (83.7)	
Positive	5 (2.5)	48 (16.3)	1.5 × 10^-6^
First-line treatment			
Surgery only	114 (57.9)	193 (65.4)	
Surgery + TACE	83 (42.1)	102 (34.6)	0.090

*P values were compared by chi-square test for categorical variables and Student’s *t* test for continuous variable.

Abbreviations: AFP, α-fetal protein; BCLC, Barcelona Clinic Liver Cancer; HBsAg, hepatitis B virus surface antigen; SD, standard deviation; PVTT, portal vein tumor thrombus; TACE, transcatheter arterial chemoembolization

### Associations between individual SNPs and RFS of HCC patients

We assessed the association of each individual SNP with the recurrence of HCC under dominant, recessive and additive models, and presented the results as best-fitting model with the smallest *P* value. As shown in Table 2, SNPs rs12064957 and rs3935401 in *SDHC* gene were significantly associated with increased HCC overall recurrence risk under recessive (HR = 2.01, 95%CI 1.02–3.96, *P* = 0.044) and additive (HR = 1.24, 95%CI 1.00–1.53, *P* = 0.049) models, respectively. All three SNPs in *SDHD* gene were significantly associated with the increased HCC recurrence risk under dominant model. However, these three SNPs in *SDHD* gene exhibited strong LD coefficient r^2^ > 0.8 (S1 Fig). Therefore, we only included the most significant SNP rs10789859 in further analyses. SNPs rs1414493 in *FH* gene and rs11540478 in *IDH2* gene were significantly associated with the decreased HCC overall recurrence risk under dominant (HR = 0.77, 95%CI 0.61–0.97, *P* = 0.030) and recessive (HR = 0.25, 95%CI 0.09–0.68, *P* = 0.007) models, respectively. We further evaluated the effects of individual SNPs on early recurrence of patients and found very similar results as the overall recurrence analysis. Furthermore, all the significant SNPs had *q* values smaller than 0.12, suggesting relatively low probability of false positive findings in our study (Table 2).

**Table 2:** Association of polymorphisms in TCA cycle genes with recurrence in HCC patients.

Genesymbol	SNP	Overall recurrence				Early recurrence				Late recurrence			
		Best fitting model	HR (95%CI)*	*P* value	q value	Best fitting model	HR (95%CI)*	*P* value	q value	Best fitting model	HR (95%CI)*	*P* value	q value
*SDHA*	rs13173911	REC	0.79 (0.45–1.40)	0.424	0.551	REC	0.86 (0.49–1.51)	0.590	0.669	NA	NA	NA	NA
	rs2864963	REC	0.70 (0.46–1.06)	0.089	0.189	REC	0.73 (0.48–1.12)	0.154	0.291	REC	0.80 (0.33–1.91)	0.614	0.878
*SDHB*	rs3754509	REC	1.11 (0.81–1.53)	0.515	0.584	REC	1.20 (0.87–1.65)	0.266	0.411	REC	1.09 (0.46–2.57)	0.853	0.910
*SDHC*	rs12064957	REC	**2.01 (1.02–3.96)**	**0.044**	0.119	REC	**2.25 (1.14–4.45)**	**0.019**	0.065	ADD	0.65 (0.22–1.95)	0.438	0.878
	rs4131826	ADD	0.92 (0.77–1.12)	0.416	0.551	ADD	0.94 (0.77–1.14)	0.502	0.656	REC	0.67 (0.26–1.72)	0.409	0.878
	rs3935401	ADD	**1.24 (1.00–1.53)**	**0.049**	0.119	ADD	**1.26 (1.01–1.56)**	**0.038**	0.100	REC	1.04 (0.42–2.61)	0.928	0.928
*SDHD*	rs544184	DOM	**1.56 (1.22–2.01)**	**4.8 × 10** ^**–4**^	**0.003**	DOM	**1.62 (1.25–2.11)**	**3.0 × 10** ^**–4**^	**0.002**	REC	1.18 (0.49–2.82)	0.710	0.878
	rs7121782	DOM	**1.59 (1.24–2.05)**	**3.0 × 10** ^**–4**^	**0.003**	DOM	**1.65 (1.27–2.15)**	**1.8 × 10** ^**–4**^	**0.002**	REC	1.18 (0.49–2.82)	0.709	0.878
	rs10789859	DOM	**1.60 (1.25–2.06)**	**2.3 × 10** ^**–4**^	**0.003**	DOM	**1.67 (1.28–2.18)**	**1.3 × 10** ^**–4**^	**0.002**	REC	1.14 (0.48–2.72)	0.768	0.878
*FH*	rs12071124	DOM	0.91 (0.70–1.17)	0.454	0.551	DOM	0.92 (0.70–1.21)	0.547	0.664	REC	0.75 (0.30–1.89)	0.548	0.878
	rs7530270	DOM	0.90 (0.69–1.16)	0.403	0.551	DOM	0.91 (0.69–1.19)	0.470	0.656	REC	0.81 (0.32–2.05)	0.655	0.878
	rs1414493	DOM	**0.77 (0.61–0.97)**	**0.030**	0.102	DOM	**0.78 (0.61–0.99)**	**0.041**	0.100	DOM	0.64 (0.27–1.56)	0.329	0.878
*IDH1*	rs12478635	DOM	0.87 (0.67–1.11)	0.263	0.447	DOM	0.84 (0.65–1.09)	0.198	0.337	REC	1.78 (0.59–5.42)	0.307	0.878
*IDH2*	rs11540478	REC	**0.25 (0.09–0.68)**	**0.007**	**0.030**	REC	**0.21 (0.07–0.67)**	**0.008**	**0.034**	REC	1.17 (0.47–2.92)	0.733	0.878
	rs4283211	DOM	0.94 (0.74–1.19)	0.604	0.604	REC	1.12 (0.71–1.77)	0.635	0.675	REC	0.44 (0.18–1.10)	0.078	0.878
	rs11632348	DOM	0.93 (0.74–1.18)	0.557	0.592	REC	1.06 (0.67–1.67)	0.819	0.819	REC	0.51 (0.20–1.26)	0.143	0.878
	rs4932158	REC	0.64 (0.36–1.12)	0.118	0.223	REC	0.61 (0.34–1.09)	0.097	0.206	REC	0.83 (0.29–2.36)	0.730	0.878

*HR was adjusted for age, gender, HBsAg status, serum AFP level, TNM stage, tumor differentiation and treatment.

Abbreviations: ADD, additive model; DOM, dominant model; REC, recessive model; TFBS, transcription factor binding site.

Significant *P* values (< 0.05) were bolded. And q values were calculated by multiple comparison q tests.

### Cumulative effects of risk genotypes on HCC recurrence

**Fig 1:**
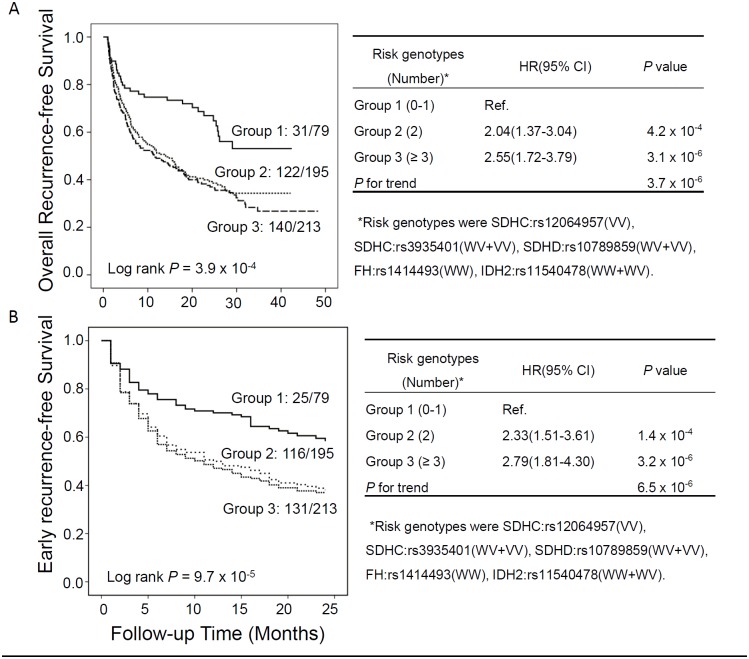
Cumulative effects of risk genotypes. A) Overall recurrence free survival; B) Early recurrence free survival. Cumulative effects of risk genotypes groups were assessed by Kaplan-Meier curves (left panels) and estimated using Cox proportional hazards model by multivariate analysis (right panels). Individual SNPs with significant *P* values <0.05 were included to categorize the risk genotype groups.

To evaluate the cumulative effect of multiple SNPs on HCC recurrence risk, we combined the risk genotypes of significant individual SNPs and analyzed the associations with HCC recurrence risk by multivariate Cox proportional hazards model. According to the significant results of best-fitting model in Table 2, the risk genotypes of 5 selected significant SNPs were defined as following: SDHC:rs12064957(VV), SDHC:rs3935401(WV+VV), SDHD:rs10789859(WV+VV), FH:rs1414493(WW), IDH2:rs11540478(WW+WV). In the overall recurrence analysis, the significantly increased recurrence risks of HCC were observed in patients with 2 risk genotypes (HR = 2.04, 95%CI 1.37–3.04, *P* = 4.2 × 10^-4^) and in patients with ≥ 3 risk genotypes (HR = 2.55, 95%CI 1.72–3.79, *P* = 3.1 × 10^-6^), when comparing to those with ≤ 1 risk genotype. The cumulative effects of combined risk genotypes exhibited a significant dose-dependent manner with *P* for trend of 3.1 × 10^-6^ (Fig 1A, right panel). Kaplan-Meier curves significantly distinguished the patients with > 1 risk genotypes from those with ≤ 1 risk genotype (log-rank *P* = 3.9 × 10^-4^) by overall recurrence-free survival analysis (Fig 1A, left panel). There were very similar results on the cumulative effects of combined risk genotypes in early recurrence-free survival analysis (Fig 1B).

### Stratified and joint analyses for cumulative effects of risk genotypes on early recurrence of HCC

We conducted the stratified analyses to assess the cumulative effects on early recurrence rate in different strata of host variables by Kaplan-Meier curves. As shown in S2 Fig, HCC patients with ≥ 2 risk genotypes had significantly higher early recurrence rates than those with < 2 risk genotypes in the strata of subgroups without TACE treatment (Log rank *P* = 2.2 × 10^-5^) and with diseases at early stages (I+II) (Log rank *P* = 7.4 × 10^-5^), but not in strata of subgroups with TACE treatment (Log rank *P* = 0.167) and with TNM stage III or IV diseases (Log rank *P* = 0.222) (S2 Fig, right panels). In addition, we further conducted the joint analyses to reveal if the major characteristics will have significant interaction with the number of risk genotypes on HCC early recurrence. As shown in S2 Table, the significant increased HCC early recurrence risk were observed in patients with the combinations of higher risk genotypes and higher serum AFP level advanced tumor stage, poor tumor differentiation, or no TACE treatment. Significant interactions were exhibited between the genetic factor and clinical parameters with *P* for interaction < 0.001.

### Stratified analysis for effect of TACE treatment on early recurrence of HCC by risk genotypes

**Fig 2:**
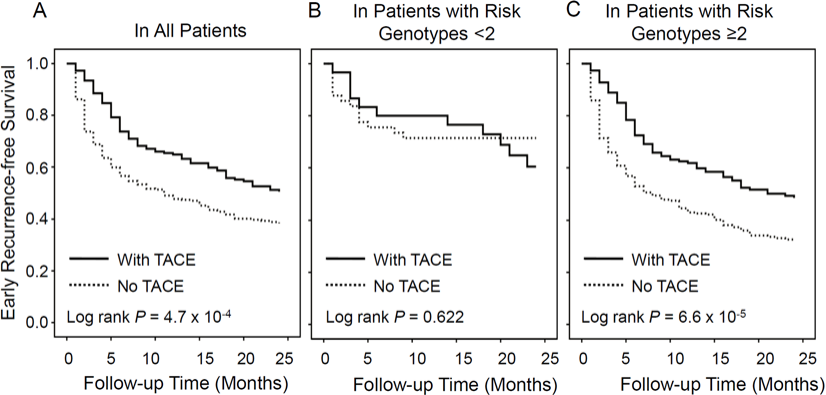
Modified effects of TACE treatment on HCC recurrence stratified by the number of risk genotypes. A) In all patients; B) In patients with less than 2 risk genotypes; C) In patients with equal to or more than 2 risk genotypes.

We further evaluated the protective effects of TACE treatment on HCC patients’ early recurrence stratified by combined risk genotypes. Kaplan-Meier analysis showed that TACE treatment significantly increased the early recurrence-free survival time in all patients with log rank *P* value of 4.7 × 10^-4^ (Fig 2A). Interestingly, there was no protective effect conferred by TACE treatment in subcohort of patients with less than 2 risk genotypes (log rank *P* = 0.622) (Fig 2B). However, the significant protective effect conferred by TACE treatment was remained in subcohort of patients with equal to or more than 2 risk genotypes (log rank *P* = 6.6 × 10^-5^) (Fig 2C).

### Higher order gene-gene interactions and early recurrence of HCC

**Fig 3:**
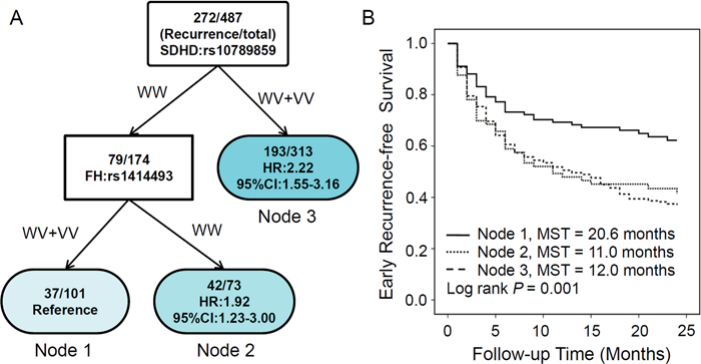
Potential higher order gene—gene interactions among TCA pathway polymorphisms. A) Tree structure identifying subgroups of patients with different genetic backgrounds; B) Kaplan—Meier survival curves for patients based on survival tree analysis.

We explored the higher order gene-gene interactions to reveal whether complex interactions among these SNPs could potentially predict the patient’s recurrence by survival tree analysis. Among 7 significant SNPs in the early recurrence analysis, we identified 2 SNPs, SDHD:rs10789859 and FH:rs1414493, which resulting in 3 terminal nodes with different early RFS time (Fig 3). The initial split on the survival tree was due to SDHD:rs10789859 (Node 3), suggesting that this SNP was the primary risk factor contributing to the RFS difference in this HCC population. Patients in the reference group (Node 1) were composed of individuals with 2 favorable genotypes, including wild-type of SDHD:rs10789859 and variant-containing genotypes of FH:rs1414493. Comparing to the reference group, patients carrying wild-type of FH:rs1414493 (Node 2) and variant-containing genotypes of SDHD:rs10789859 (Node 3) exhibited significant increased early recurrence risk with HR of 1.92 (95%CI 1.23–3.00) and 2.22 (95%CI 1.55–3.16), respectively (Fig 3A). Kaplan-Meier curve analysis also indicated the significantly decreased early RFS time in Node 2 and Node 3 patients, comparing to the reference group (Node 1), with log rank *P* value of 0.001 (Fig 3B).

Furthermore, we separated the primary cohort (n = 487) to FMMU cohort (n = 190) and SMMU cohort (n = 297) based on the hospital sites, and validated the survival tree analysis results by grouping the patients according to the survival tree model using multivariate Cox proportional hazard regression model. Comparing to node 1 patients, the significant or borderline significant higher recurrence risks were still observed in node 2 and node 3 patients for both sub-cohort analyses. The significant *P* for trend tested in our survival tree models suggested a potential clinical significance of our study for predicting early HCC recurrence (S3 Table).

## Discussion

In the present study, we evaluated the associations of potential functional SNPs in the genes encoding TCA cycle key enzymes (*SDH*, *FH* and *IDH*) with the recurrence of HCC. Our study identified 7 SNPs in *SDHC*, *SDHD*, *FH*, and *IDH2* genes to be significantly associated with HCC overall recurrence and early recurrence. Significant cumulative effects by a combination of significant individual risk genotypes were observed in overall and early recurrence analyses. Further stratified analysis suggested that the number of risk genotypes may modify the protective effect conferred by TACE treatment. Finally, the survival tree analysis revealed that SNP rs10789859 in *SDHD* gene was the primary factor contributing to HCC recurrence in our population. To the best of our knowledge, our study for the first time reported the association between SNPs in genes of TCA cycle key enzymes and HCC recurrence risk.

It is well recognized that the alteration of cellular metabolism is a hallmark of cancer, contributing to malignant transformation and to the growth and maintenance of tumors [13]. The fundamental physiological roles of SDH, FH, and IDH as TCA cycle key metabolic enzymes are to catalyze the oxidation of succinate to fumarate, hydration of fumarate to L-malate, and oxidative decarboxylation of isocitrate to α-ketoglutarate (α-KG) and CO_2_ in mitochondria, respectively. A lot of studies have suggested that loss-of-function mutations in SDH complex and FH lead to accumulation of omcometabolites such as succinate and fumarate, and oncogenic mutations of IDH produces the oncometabolite 2-hydroxyglutarate (2-HG), which provides strong evidence to link abnormal metabolism with cancers [8,14–18]. In our study, 5 significant SNPs were located in the predicted functional transcript factor binding sites of the corresponding genes, 1 SNP at the potential miRNA binding site, and 1 SNP in mRNA splicing site, suggesting that these genetic variations may influence the expression level of the metabolic enzymes, and thus alter the metabolite profiling and contribute to HCC progression. In addition to amino acid changes by SNPs in genes’ coding regions, SNPs in non-coding regions also directly alter gene functions through various mechanisms such as changing transcription activity, mRNA processing, and the affinity of miRNA binding activity [19]. However, specific mechanisms by which the significant SNPs affect the enzyme expression and functions are needed to be further investigated.

Although surgical resection is the most effective curative treatment for HCC and markedly improves the prognosis of HCC patients, the high recurrence rate (50%-70% at 5 years) remains a major challenge in HCC therapy [3,20]. Early HCC recurrence defined as recurrence within 2 years after surgery is mainly related to local invasion and intrahepatic metastasis; whereas late recurrence, occurring beyond 2 years after surgery, is mainly related to *de novo* tumor formation [21,22]. It has been reported that the intrahepatic recurrence accounts for 70%-100% of the recurrent cases after surgical resection [23,24]. In our study, we demonstrated that SNPs in genes encoding TCA cycle key enzymes were significantly associated with early HCC recurrence. More interestingly, the stratified analysis showed that the loco-regional therapy of TACE had significantly improved early HCC recurrence free survival in patients carrying with no less than 2 risk genotypes, but no significant result was observed in patients with less than 2 risk genotypes. Concordantly, our previous studies have identified some single genetic polymorphisms which are significantly associated with TACE treatment response in unresectable HCC patients [25,26]. Together, our study suggests that it may have clinical significance to incorporate these SNPs into the current tumor staging systems for the prognosis assessment and tailored treatment decision of HCC patients, once our finding is validated. In addition, we did not identify any SNP to be associated with late HCC recurrence. However, we cannot rule out the possibility of false negative results due to the small number of late recurrence events (n = 21) in our population. Further studies with larger population are warranted to investigate the association between the SNPs in genes encoding TCA cycle key enzymes and HCC late recurrence.

Cancer is a complex disease involved by multiple factors and their interactions could affect the clinical outcomes [27]. Therefore, we conducted survival tree analysis to observe the higher order gene-gene interactions among SNPs in genes encoding TCA cycle key enzymes and their effects on early recurrence in HCC patients. We found that SNP rs10789850 in *SDHD* gene as the primary split in the survival tree analysis exhibited the strongest influence on HCC patients’ early recurrence risk. Human *SDHD* gene encodes the small subunit of cytochrome b in mitochondrial succinate-ubiquinone oxidoreductase [28]. Mutations in *SDHD* gene has been initially reported to be associated with hereditary paraganglioma and *SDHD* has been suggested to play a role as tumor suppressor [29]. Subsequently, the reduced protein expression and loss of heterozygosity of *SDHD* gene have been identified in colorectal and gastric cancers [30]. In comparison, our study for the first time reported that SNPs in *SDHD* gene are associated with HCC prognosis. However, the underlying mechanism of how SDHD plays a role in HCC recurrence needs to be further examined.

## Conclusions

Our study demonstrates that genetic variations in genes encoding TCA cycle key enzymes are significantly associated with the early recurrence of HCC after surgical resection in a hospital-based Chinese patient cohort. Further functional studies are warranted to reveal the underlying mechanism.

## Supporting Information

S1 Fig Heatmap of linkage disequilibrium (LD) among the selected SNPs. The red box indicated a strong LD coefficient of r^2^>0.8.
(TIF)

S2 Fig Association of risk genotypes and HCC early recurrence by stratified analysis. A)Stratified by TACE treatment, B) Stratified by tumor stage.
(TIF)

S1 Table Information of SNPs and genotyping results. (XLS)

S2 Table Association of risk genotypes with early recurrence in HCC patients stratified by patients’ characteristics. (XLS)

S3 Table Validation of survival tree analysis in early recurrence HCC. (XLS)
