# Supplementary figures and images for: Polymorphisms in Genes of Tricarboxylic Acid Cycle Key Enzymes Are Associated with Early Recurrence of Hepatocellular Carcinoma
Source: PLoS One. 2015 Apr 20;10(4):e0124471. doi: 10.1371/journal.pone.0124471 (PMC4404327; doi:10.1371/journal.pone.0124471)

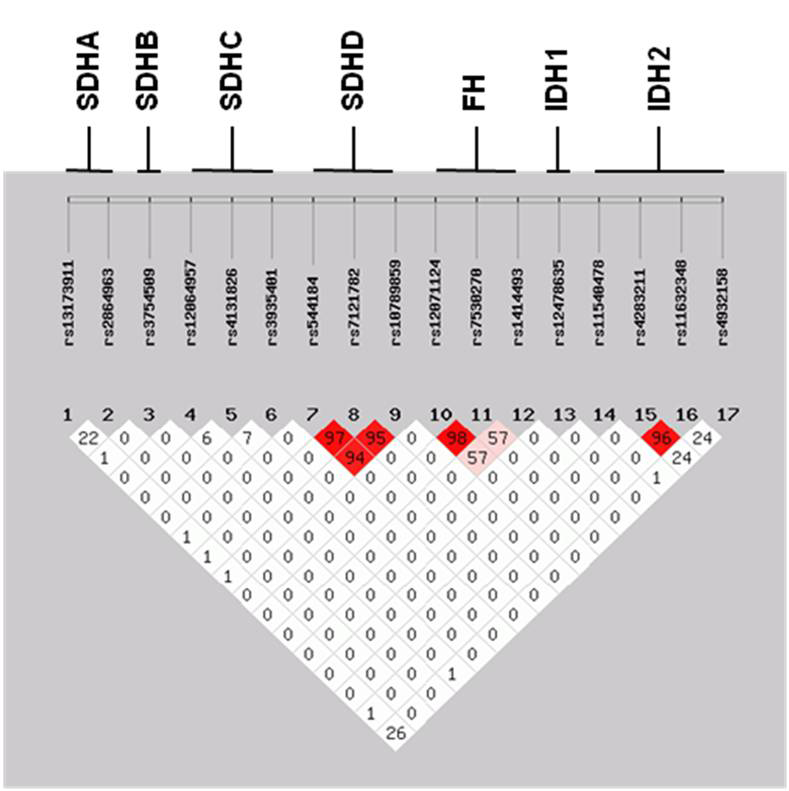

Supplement: S1 Fig — The red box indicated a strong LD coefficient of r2>0.8. (TIF) [file pone.0124471.s001.tif]

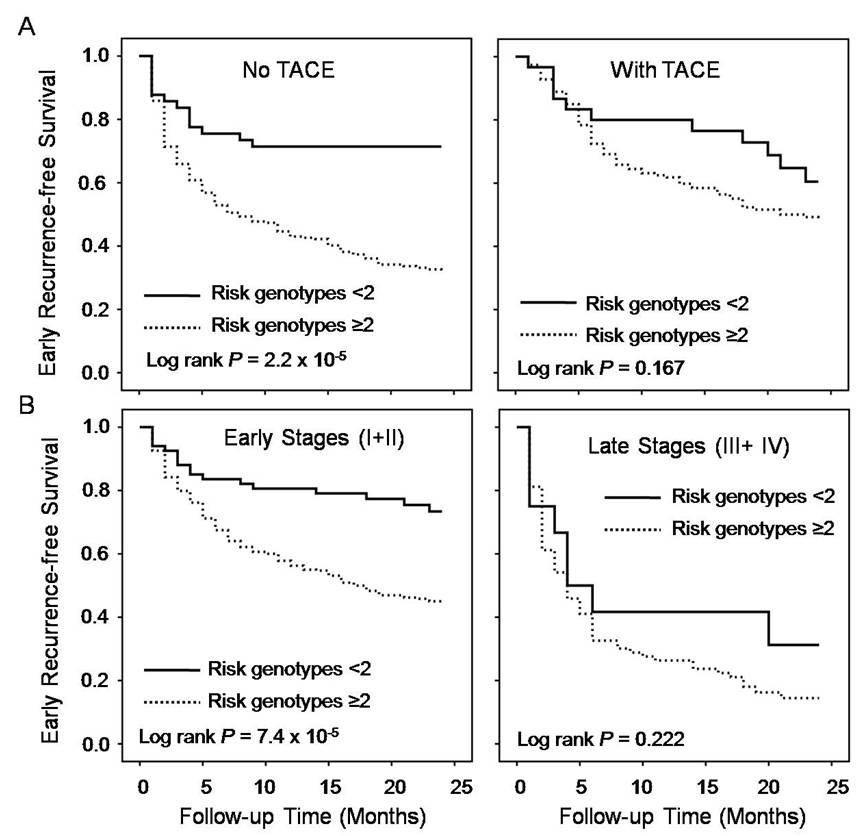

Supplement: S2 Fig — A)Stratified by TACE treatment, B) Stratified by tumor stage. (TIF) [file pone.0124471.s002.tif]
